# Associations between Fruit Mineral Composition and Polyphenol Profiles across 140 Dessert Apple Cultivars (*Malus domestica*)

**DOI:** 10.1021/acs.jafc.6c06394

**Published:** 2026-07-15

**Authors:** Eleonora Zickenheiner

**Affiliations:** 9373 Humboldt-Universität Zu Berlin, Faculty of Life Sciences Albrecht Daniel Thaer-Institute of Agricultural and Horticultural Sciences, Invalidenstraße 42, Berlin 10115, Germany

**Keywords:** *Malus domestica*, mineral−polyphenol associations, cultivar diversity, partial least-squares, generalized additive models, sulfur, chlorogenic acid

## Abstract

Apple polyphenols are key determinants of fruit quality and potential health-related properties, yet their association with fruit mineral composition remains insufficiently characterized at the cultivar scale. We quantified five minerals (K, S, P, Mg, Ca) by ICP-OES and 13 polyphenol variables by HPLC-MS across 140 dessert apple cultivars (*Malus domestica*) grown under common conditions. FDR-corrected Pearson correlations revealed selective, compound-specific patterns, with potassium and phosphorus showing the strongest positive associations with chlorogenic acid (K: r = 0.55, q = 1.2 × 10^–10^; P: r = 0.43, q = 2.7 × 10^–6^), both robust under Spearman analysis. Partial correlations identified potassium as the dominant independent correlate (partial r = 0.39, q = 6.5 × 10^–5^), while phosphorus, sulfur, and magnesium associations reflected collinearity with potassium. PLS showed only modest coupling (Q^2^ ≈ 0.06, R^2^Y ≈ 0.13). Exploratory sulfur–chlorogenic acid and calcium–cyanidin patterns remain preliminary; findings represent cultivar-scale associations, not orchard-management recommendations.

## Graphical abstract

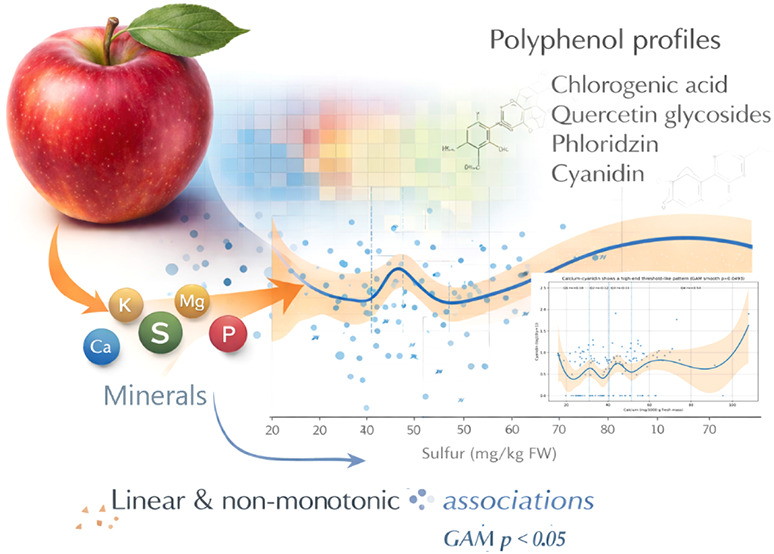

## Introduction

Polyphenols contribute substantially to apple fruit quality (color, astringency, browning behavior) and are widely studied in nutritional and plant physiological contexts. Their biosynthesis is rooted in the phenylpropanoid pathway and is shaped primarily by genotype, but also by developmental stage and environmental conditions.^1–3^

Besides light and temperature, mineral nutrition can modulate carbon allocation, redox balance, enzyme activity, and stress signaling—processes that plausibly influence phenylpropanoid flux. Potassium (K) affects osmoregulation and phloem transport, phosphorus (P) is central to energy metabolism, magnesium (Mg) underpins photosynthetic carbon assimilation, calcium (Ca) is involved in signaling and cell wall structure, and sulfur (S) connects to amino acid and antioxidant metabolism (e.g., glutathione).^4,5^

Previous work has characterized apple phenolics, minerals, or both, but the statistical integration of fruit mineral status with a multicompound polyphenol matrix remains limited. Starowicz et al. summarized the diversity and physiological relevance of phenolic compounds in apple, but their review did not address mineral–polyphenol coupling at cultivar scale.^6^ Preti and Tarola profiled ancient apple cultivars from Northeast Italy for polyphenols, antioxidant capacity and minerals, providing important compositional reference data but without focusing on nonlinear mineral–phenolic association modeling.^7^ Cice et al. recently combined polyphenolic and mineral profiling in autochthonous apple varieties, further demonstrating the value of integrated compositional data sets; however, the extent to which individual fruit minerals covary with specific phenolic subclasses across a broad common-garden cultivar panel remains insufficiently resolved.^8^ Earlier apple-specific work by Awad and de Jager already documented that fruit N, P, K, Mg and Ca concentrations differentially correlate with skin flavonoid and chlorogenic acid levels within single cultivars,^24^ providing the immediate precedent for the broader common-garden approach taken here.

The rationale of the present study was therefore not to infer direct nutritional or plant-protection effects, but to test whether cultivar-level variation in fruit mineral composition carries measurable information about phenolic profiles under reduced environmental heterogeneity. Such information is relevant for interpreting cultivar comparisons because mineral status may act as a covariate or modifier when comparing phenolic composition among genetically diverse cultivars. It may also help identify mineral–phenolic pairs that merit controlled intervention experiments, while avoiding causal interpretation from observational data.

The objectives were: (i) to quantify the cultivar-scale distribution of five fruit minerals and 13 polyphenol variables in a common-garden panel of 140 dessert apple cultivars; (ii) to evaluate mineral–polyphenol associations using Pearson correlations with FDR correction and Spearman rank sensitivity analyses; (iii) to describe the internal correlation structure within the mineral and polyphenol blocks; (iv) to assess the extent of multivariate coupling between the mineral and polyphenol matrices using PLS; and (v) to explore selected candidate nonlinear patterns using spline/GAM visualization and robustness checks. The novelty of the study lies in combining broad cultivar diversity, common-garden sampling, mineral profiling and multicompound polyphenol profiling with both linear and exploratory nonlinear association analyses, while explicitly separating hypothesis generation from causal orchard-management inference.

## Materials and Methods

### Plant Material and Sampling

Scionwood for grafting was obtained in January 2019 from variety conservation centers with genetically verified cultivar identity. Trees were grown under a documented organic orchard-management routine in the district of Lörrach (Baden-Württemberg, Germany). All cultivars were grafted on M9 rootstocks and received the same site-level cultivation program. Plant protection included the use of wettable sulfur (Kumulus WG) as part of the organic scab-management routine, and calcium was supplied as a foliar fertilizer (Lebosol-Calcium) for bitter-pit prevention.^9–11^ These inputs were applied as part of the uniform orchard-management regime and were not experimentally varied among cultivars or treatment groups. Therefore, the study should be interpreted as a common-garden cultivar comparison under documented organic cultivation conditions, not as a sulfur- or calcium-intervention trial.

For each cultivar, nine fully ripe and healthy fruits (dessert apple quality) were harvested from three trees (three fruits per tree). From each fruit, 30 g of edible tissue (flesh with peel; excluding the core) was collected as wedge sections sampled from both the sun-exposed and shaded sides. The resulting composite sample (270 g edible portion per cultivar) was briefly rinsed under cold running tap water to remove surface contamination (e.g., soil dust, sand and bird droppings) and gently blotted dry with paper tissue to avoid sample dilution, so that fruits were clean and surface-dry before processing. The composite was immediately frozen (−80 °C) and lyophilized. Lyophilized apple material was pulverized using a Retsch Knife Mill GM300. Because freeze-dried apple tissue is hygroscopic, milling was performed at high speed for short intervals to minimize moisture uptake from ambient air and to limit heat buildup during grinding. Milling was performed to obtain a visually homogeneous powder suitable for representative subsampling. Subsampling material was then milled to a fine powder using a Retsch MM 400 Vibrating Mill (27 Hz, 1.30 min). The average particle size was not determined by sieving or laser diffraction; the homogenate was vacuum-sealed immediately after milling and stored at −80 °C until extraction and analysis. Because one composite was generated per cultivar, the cultivar-level composite—not individual fruits or trees—was the statistical unit for association analyses.

### Mineral and Polyphenol Analysis

Mineral elements (K, S, P, Mg, Ca) were quantified by inductively coupled plasma–optical emission spectrometry (ICP-OES, iCAP 6300 Duo MFC, Thermo Scientific) from acidified aqueous digests using a plant/fruit matrix digestion workflow adapted from established mineral-analysis approaches.^4,12^ Freeze-dried apple samples were rehomogenized, and two analytical subsamples per cultivar (A/B; duplicate determination from the same cultivar homogenate) were weighed (0.25 g; mass correction applied) into microwave-transparent CEM digestion tubes. Samples were digested with 5 mL of 65% HNO_3_ and 3 mL of 30% H_2_O_2_ using a microwave program of 800 W for 20 min to 200 °C (5 min ramp), followed by 800 W for 1 min to 210 °C (5 min ramp) and 800 W for 1 min to 220 °C (5 min ramp), then cooled for 30 min. Digests were quantitatively transferred to volumetric flasks and brought to 50 mL with distilled, demineralized water, then filtered to protect the ICP torch. Element concentrations were determined based on element-specific emission lines and external calibration. In parallel, dry matter was determined by drying 2.0 g of milled sample at 105 °C for 3 h; mineral contents were calculated on a dry-matter basis and subsequently back-calculated to fresh-mass equivalents. Analytical precision was assessed from duplicate digestion/measurement and quality-control checks; deviations were <3%. This precision statement refers to analytical repeatability and does not quantify biological variability among trees or fruits.

Thirteen polyphenol variables were extracted from freeze-dried apple material and quantified by HPLC-MS: chlorogenic acid, p-Coumaroyl-quinic acid, quercetin-3-glucoside, quercetin-3-xyloside, quercetin-3-pentoside, catechin, epicatechin, procyanidin dimer, procyanidin trimer, procyanidin tetramer, phloridzin-2-xylosyl-glucoside, phloridzin-2-glucoside, and cyanidin. Polyphenols were extracted and quantified in duplicate for each cultivar from analytical subsamples A and B of the same homogenized cultivar composite. For each subsample, 20 mg of freeze-dried, finely ground material was weighed into a reaction tube and extracted with 600 μL of 60% methanol. Samples were vortexed and shaken for 40 min at 1400 rpm and 20 °C, then centrifuged at 4500 rpm and 20 °C for 15 min. The supernatant was transferred to a fresh tube and stored at 4 °C. The remaining pellet was re-extracted twice with 300 μL of 60% methanol each. Combined extracts were evaporated to complete dryness in a vacuum centrifuge and the residue was reconstituted in 200 μL of 10% methanol. The solution was clarified using Spin-X filter tubes (3000 rpm, 5 min, 20 °C) and transferred to autosampler vials. Polyphenols were quantified by HPLC-MS using a previously published apple polyphenol profiling method.^13,14^ Chromatographic separation was performed on a reversed-phase C18 column as described in these references. The mobile phases, gradient program, flow rate, and MS detection parameters (including ionization mode and mass transitions) followed the published method. In the present study, quantification was based on ten-point external calibration curves prepared from authentic reference standards for the target compounds, and samples were injected in randomized order. Polyphenol concentrations were calculated as mg kg^–1^ FW after conversion from freeze-dried material to fresh-weight equivalents.

### Statistical Analysis

Correlation Analysis: Distributions were summarized per variable (mean, SD, median, range, skewness and zero counts where applicable). Associations between minerals and polyphenols were evaluated using Pearson correlations (r) across the 5 × 13 mineral–polyphenol matrix and corresponding p-values. Benjamini–Hochberg FDR correction was applied as the primary multiple-testing criterion for this matrix, and FDR-adjusted q-values are reported alongside nominal p-values where relevant.^15^ Because several variables were right-skewed or zero-inflated, Spearman rank correlations (ρ) were calculated as a sensitivity analysis. In addition, within-block correlation matrices were calculated for the five minerals and for the 13 polyphenol variables to describe collinearity within predictor and response blocks; these results are provided in the Supporting Information. To assess the independent contribution of individual minerals given their mutual collinearity, partial correlations between each mineral and each polyphenol were computed, controlling for the remaining four minerals (linear residualization), and ridge regression (scikit-learn; autoscaled data; penalty α = 1.0) was used as a complementary multivariate check.

PLS Regression: To integrate mineral composition (predictor set) with the multicompound polyphenol matrix (response set), we performed partial least-squares regression (PLS) using the five quantified minerals as X variables and the 13 quantified polyphenol variables as Y variables (n = 140 cultivars).^16^ Prior to modeling, X and Y were autoscaled (mean-centered and scaled to unit variance). The number of latent components was evaluated by 10-fold cross-validation. Model performance was summarized by cross-validated Q^2^, RMSECV, the fitted-model R^2^Y, and the cumulative percentage of X-variance captured by the selected component structure. Variable importance in projection (VIP) scores were calculated from the selected three-component model; given the low Q^2^/R^2^Y, VIP values were used only for within-model prioritization and not as evidence of strong predictive control.

Exploratory spline/GAM analysis: Generalized additive models were fitted in Python/Google Colab using statsmodels GLMGam as exploratory spline-based models for selected mineral–polyphenol relationships.^17–19^ Polyphenol concentrations were log_10_(concentration +1)-transformed prior to modeling to reduce skewness and accommodate zero values, and mineral predictors were z-standardized. Cubic B-splines with a basis dimension of k = 10 were used, and the smoothing penalty was selected by generalized cross-validation. Because the GAMs were implemented in Python (statsmodels GLMGam) rather than in R/mgcv, the mgcv-specific k-index diagnostic was not directly available. However, the effective degrees of freedom of both fitted smooths were well below the maximum permitted by the chosen basis (k – 1 = 9): edf = 1.61 for the sulfur–chlorogenic acid smooth and edf = 0.79 for the calcium–cyanidin smooth. The smooths were therefore strongly penalized rather than basis-limited, providing no indication that the chosen basis dimension was inadequate. Smooth-term significance was assessed by approximate Wald-type tests with FDR correction across the two reported smooths, and patterns were interpreted descriptively in conjunction with FDR-corrected Pearson/Spearman correlations and robustness checks. Given the exploratory objective and the observational common-garden design, interpretation focused on candidate pattern shape and robustness rather than on causal inference.

Statistical analyses were performed in Python 3.13.5 using NumPy 2.3.5, pandas 2.2.3, SciPy 1.17.0, scikit-learn 1.8.0 and statsmodels 0.14.6 for correlation analysis, PLS regression, exploratory GAM/spline modeling and sensitivity analyses.

### Safety Considerations

*Concentrated nitric acid (65% HNO*_*3*_ *) and hydrogen peroxide (30% H*_*2*_ *O*_*2*_ *) used in the microwave digestion procedure are strong oxidizers and corrosive. All digestion work was performed in a fume hood by trained personnel wearing appropriate personal protective equipment (safety goggles, nitrile gloves, lab coat). Microwave digestion vessels were operated within manufacturer-specified pressure and temperature limits, and vessels were vented and cooled before opening. No other unusual hazards are noted.*

## Results and Discussion

### Mineral and Polyphenol Contents across 140 Cultivars

Descriptive statistics for all analytes are presented in Table 1 and visualized in Figure 1. Across cultivars, polyphenol concentrations varied widely and were generally right-skewed, consistent with a few high-accumulating cultivars for several compounds. Chlorogenic acid averaged 225.4 ± 150.9 mg kg^–1^ FW (range 21.0–892.3), and catechin averaged 343.2 ± 153.4 mg kg^–1^ FW (range 116.9–857.0). Variability was particularly pronounced for cyanidin (CV 177%; 46/140 cultivars with zero values) and p-Coumaroyl-quinic acid (CV 101%), indicating strong cultivar dependence.

**Table 1:** Summary Descriptive Statistics of 13 Polyphenols and 5 Mineral Elements in 140 Apple Cultivars Grown under Identical Orchard Conditions^a^ ^b^

Analyte	Mean	SD	CV (%)	Median	Q1	Q3	IQR	Min	Max	Range	Skewness
Chlorogenic acid	225.39	150.92	66.96	184.88	124.18	293.81	169.63	20.99	892.33	871.33	1.54
p-Coumaroyl-quinic acid	76.02	76.56	100.71	59.79	32.28	96.39	64.11	4.47	603.09	598.62	3.29
Quercetin-3-glucoside	7.57	4.64	61.25	6.40	4.62	8.73	4.12	2.43	37.41	34.99	2.74
Quercetin-3-xyloside	10.93	7.02	64.18	9.33	6.37	12.94	6.57	2.39	56.68	54.29	2.63
Quercetin-3-pentoside	11.16	7.52	67.41	8.68	6.47	12.90	6.43	2.63	41.64	39.01	1.78
Catechin	343.15	153.35	44.69	316.08	239.16	403.35	164.19	116.88	856.99	740.11	1.20
Epicatechin	230.99	154.29	66.79	193.02	152.63	260.20	107.57	28.94	989.44	960.50	2.76
Procyanidin Dimer	209.86	88.97	42.40	197.34	151.23	255.22	104.00	12.62	493.94	481.32	0.67
Procyanidin Trimer	124.93	72.05	57.67	116.47	75.23	164.05	88.81	0.00	356.57	356.57	0.67
Procyanidin Tetramer	105.02	61.78	58.83	93.98	60.60	138.05	77.45	9.63	388.91	379.28	1.53
Phloridzin-2-xylosyl-glucoside	113.03	110.87	98.09	74.04	41.34	141.65	100.31	5.71	668.28	662.57	2.25
Phloridzin-2-glucoside	102.50	81.90	79.90	84.85	47.21	124.67	77.46	7.00	446.10	439.11	2.01
Cyanidin	6.13	10.86	177.08	4.86	0.00	7.26	7.26	0.00	80.74	80.74	5.15
Potassium	1474.92	355.24	24.09	1410.45	1236.45	1689.78	453.33	930.50	2854.80	1924.30	0.95
Sulfur	46.87	12.61	26.90	44.50	39.15	53.15	14.00	22.50	104.00	81.50	1.20
Phosphorus	124.79	35.64	28.56	117.55	102.05	139.88	37.83	50.80	255.20	204.40	1.12
Magnesium	58.95	10.03	17.01	57.85	53.38	64.73	11.35	32.00	108.90	76.90	0.87
Calcium	42.51	15.62	36.73	40.55	31.08	51.35	20.27	16.00	107.80	91.80	1.06

a Polyphenols and minerals were quantified by HPLC-MS and ICP–OES, respectively. Values are expressed as mg kg^–1^ fresh weight (FW). Reported are mean, SD, median, Q1–Q3 (interquartile range 25%, 75%, IQR), min–max (range), coefficient of variation (CV %), and skewness across cultivars (per-cultivar valuesrepresent means of duplicate determinations, A/B).

b Descriptive statistics (polyphenols and minerals, mg/1000 g FW).

**Figure 1:**
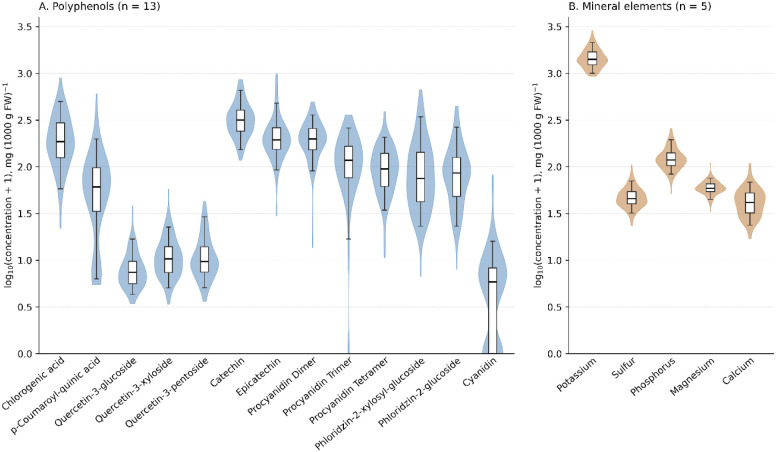
Distribution of concentrations for 13 polyphenols (A) and 5 mineral elements (B) across 140 apple cultivars. Values are shown as violin plots (kernel density) with an overlaid boxplot (median and interquartile range, IQR); whiskers indicate the fifth–95th percentiles. The *y*-axis is scaled as log_10_(x + 1) to accommodate the wide concentration range and zero values. Concentrations are expressed as mg per 1000 g fresh weight (FW).

In contrast, minerals showed comparatively lower dispersion (CV roughly 17–37%), with potassium dominating the mineral profile (mean 1474.9 ± 355.2 mg kg^–1^ FW; range 930.5–2854.8). Sulfur averaged 46.9 ± 12.6 mg kg^–1^ FW (range 22.5–104.0). Overall, under common orchard conditions, polyphenol profiles varied more strongly across cultivars than mineral concentrations. This pattern is consistent with strong cultivar dependence of phenolic metabolism, but the present design does not quantify genotype-versus-environment variance components; therefore, genotypic control is interpreted as a plausible explanation rather than as a variance-partitioning result.

### Internal Correlation Structure of Minerals and Polyphenols

The five minerals were not statistically independent. Potassium, phosphorus and magnesium formed a positively correlated mineral block (K–P r = 0.79; K–Mg r = 0.74; P–Mg r = 0.62), and sulfur correlated with magnesium (r = 0.73), potassium (r = 0.60) and phosphorus (r = 0.58). Calcium was comparatively independent from the K/P/S block (Ca–K r = 0.16; Ca–P r = −0.05; Ca–S r = 0.01), with only a weak positive relationship to magnesium (r = 0.24). The polyphenol matrix also showed strong internal structure, especially among quercetin glycosides (quercetin-3-glucoside with quercetin-3-xyloside: r = 0.95; with quercetin-3-pentoside: r = 0.83), between the two phloridzin derivatives (r = 0.68), and among flavan-3-ol/procyanidin variables. These within-block correlations support the use of multivariate description, but they also reinforce that PLS/VIP patterns must be interpreted in the context of collinearity. Full Pearson and Spearman matrices are provided in Supplementary Tables S1 and Table 2.

**Table 2:** Summary of the Main Mineral–Polyphenol Correlation Robustness Results across 140 Apple Cultivars

Mineral	Polyphenol	Pearson r	Pearson p	Pearson FDR q	P-FDR sig	Spearman rho	Spearman p	Spearman FDR q	S-FDR sig	Interpretation
**K**	Chlorogenic acid	+0.550	1.89 × 10^–12^	1.22 × 10^–10^	**yes**	+0.422	2.02 × 10^–7^	1.31 × 10^–5^	**yes**	Robust positive association
**P**	Chlorogenic acid	+0.434	8.21 × 10^–8^	2.67 × 10^–6^	**yes**	+0.344	3.23 × 10^–5^	5.24 × 10^–4^	**yes**	Robust positive association
**K**	p-Coumaroyl-quinic acid	+0.391	1.84 × 10^–6^	3.99 × 10^–5^	**yes**	+0.243	0.004	0.025	**yes**	Robust but weaker association
**P**	p-Coumaroyl-quinic acid	+0.268	0.001	0.009	**yes**	+0.289	5.27 × 10^–4^	0.004	**yes**	Robust but moderate association
**S**	Chlorogenic acid	+0.212	0.012	0.038	**yes**	+0.114	0.179	0.332	**no**	Pearson-only; not rank-robust
**Ca**	Cyanidin	+0.319	1.22 × 10^–4^	0.002	**yes**	+0.187	0.027	0.083	**no**	Attenuated in Spearman; zero-inflation sensitive
**Ca**	p-Coumaroyl-quinic acid	+0.222	0.008	0.030	**yes**	+0.035	0.679	0.849	**no**	Pearson-only; not rank-robust
**Ca**	Phloridzin-2-glucoside	+0.217	0.010	0.034	**yes**	+0.120	0.159	0.324	**no**	Pearson-only; not rank-robust
**Mg**	Chlorogenic acid	+0.302	2.92 × 10^–4^	0.002	**yes**	+0.207	0.014	0.055	**no**	Borderline; not rank-robust

### Correlation Analysis

To explore potential coupling between fruit mineral composition and polyphenol accumulation, we computed pairwise Pearson correlations between the five minerals and 13 polyphenol variables (Figure 2). FDR-adjusted q-values were used as the primary criterion for statistical interpretation. Overall, mineral–polyphenol associations were selective and compound-dependent. The strongest positive relationships were observed for potassium and phosphorus with chlorogenic acid (K: r = 0.55, p = 1.9 × 10^–12^, q = 1.2 × 10^–10^; P: r = 0.43, p = 8.2 × 10^–8^, q = 2.7 × 10^–6^). Potassium was also associated with p-Coumaroyl-quinic acid (r = 0.39, q = 4.0 × 10^–5^) and quercetin glycosides (typically r ≈ 0.31–0.34, q < 0.002), whereas phosphorus showed weaker but FDR-supported relationships with several quercetin and procyanidin variables. Magnesium showed comparatively weaker relationships; its Pearson correlation with chlorogenic acid survived FDR correction (r = 0.30, q = 0.002), whereas some additional Mg associations were not rank-robust. Sulfur showed only a weak Pearson association with chlorogenic acid (r = 0.21, p = 0.012, q = 0.038), and this association did not persist in the Spearman sensitivity analysis.

**Figure 2:**
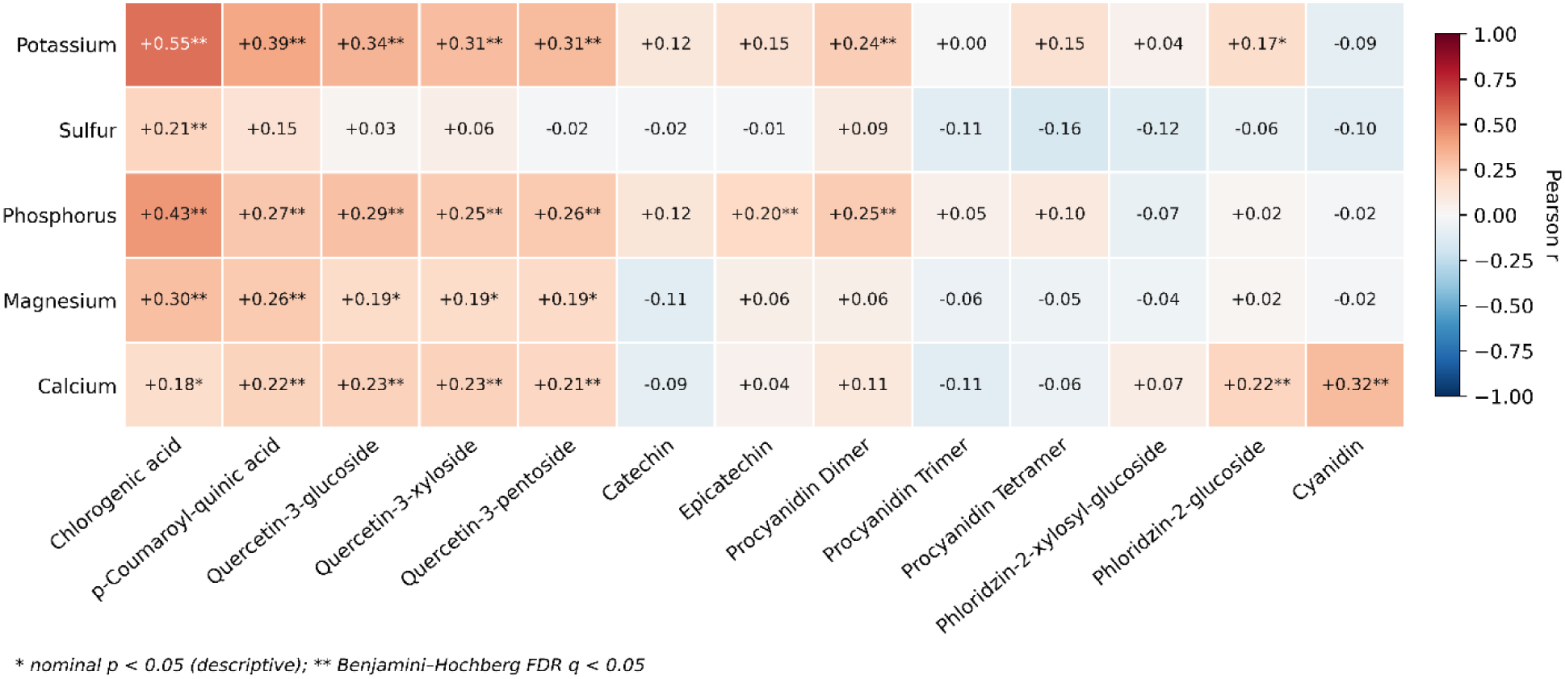
Heatmap of Pearson correlation coefficients (r) between five mineral elements (K, S, P, Mg, Ca) and 13 quantified polyphenol variables across 140 apple cultivars. Cell color indicates correlation direction and magnitude (scaled from −1 to +1). A single asterisk indicates a nominal association (* *p* < 0.05) and is shown descriptively; double asterisks indicate associations that passed the primary Benjamini–Hochberg FDR criterion (** *q* < 0.05).

Spearman correlations provided a more conservative robustness check, with 13/65 mineral–polyphenol associations passing FDR *q* < 0.05. The strongest K/P–chlorogenic acid associations remained robust, although with lower effect sizes (K: ρ = 0.42, q = 1.3 × 10^–5^; P: ρ = 0.34, q = 5.2 × 10^–4^). In contrast, sulfur–chlorogenic acid was not rank-robust (ρ = 0.11, p = 0.179), and calcium–cyanidin attenuated to a nominal but non-FDR-confirmed Spearman result (ρ = 0.19, p = 0.027, q = 0.083). Thus, the correlation analysis supports robust monotonic coupling primarily for the K/P–chlorogenic acid and K/P–quercetin-glycoside patterns, whereas sulfur and calcium patterns should be treated as exploratory candidates.

To test whether these associations reflected independent mineral effects or the shared covariance among K, P, Mg and S, we computed partial correlations between each mineral and each polyphenol, controlling for the remaining four minerals, complemented by ridge regression on autoscaled predictors (Supplementary Table S4). After controlling for the other minerals, potassium retained a clear, FDR-significant independent association with chlorogenic acid (partial r = 0.39, q = 6.5 × 10^–5^) and p-Coumaroyl-quinic acid (partial r = 0.25, q = 0.020), whereas the apparent phosphorus (partial r = 0.08), sulfur (partial r = −0.06) and magnesium (partial r = −0.15) associations were no longer significant and were therefore largely explained by their collinearity with potassium (K–P r = 0.79; K–Mg r = 0.74). Ridge regression confirmed that potassium carried by far the largest standardized coefficient for chlorogenic acid (β = 0.64, versus ≤0.22 for the other minerals). In contrast, calcium retained an independent association with cyanidin (partial r = 0.36, q = 1.5 × 10^–4^), consistent with its comparative independence from the K/P/Mg/S block. Thus, potassium is the dominant independent correlate of chlorogenic acid and p-Coumaroyl-quinic acid, whereas the calcium–cyanidin association, although independent of mineral covariance, remains exploratory given the zero-inflated cyanidin distribution (see below).

### Multivariate Patterns (PLS)

Because mineral and polyphenol traits are intercorrelated, we used PLS to evaluate multivariate structure. Cross-validation indicated modest but nonzero multivariate coupling, with the best overall predictive performance at three components (Q^2^ = 0.058; RMSECV = 0.971 in standardized units (autoscaled data)). The fitted three-component model explained 12.6% of total variance in the autoscaled polyphenol matrix (R^2^Y = 0.126) while capturing 92.0% of the variance in the autoscaled mineral predictor matrix (R^2^X = 0.920). This imbalance indicates that the five-mineral matrix is internally well summarized by the PLS components, but that it explains only a limited fraction of the polyphenol matrix. VIP scores from the three-component model were highest for calcium (VIP = 1.12) and potassium (VIP = 1.11), followed by sulfur (VIP = 1.01), whereas phosphorus (VIP = 0.90) and magnesium (VIP = 0.83) contributed less strongly within this low-performing model (Figures 3–5).

**Figure 3:**
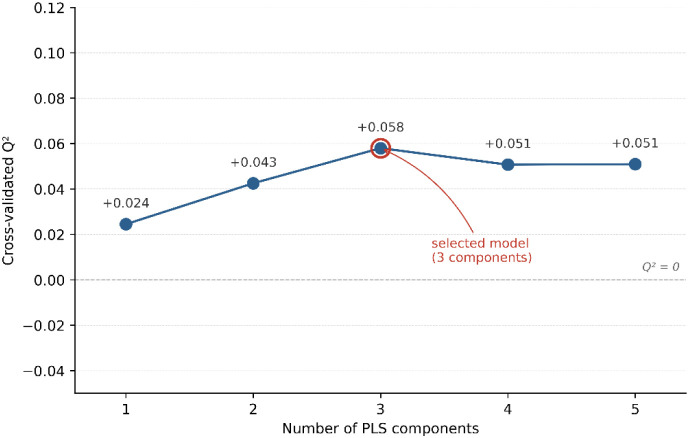
Cross-validated Q^2^ for PLS models (1–5 components) linking five minerals to the 13-compound polyphenol matrix across 140 cultivars (10-fold CV).

**4:**
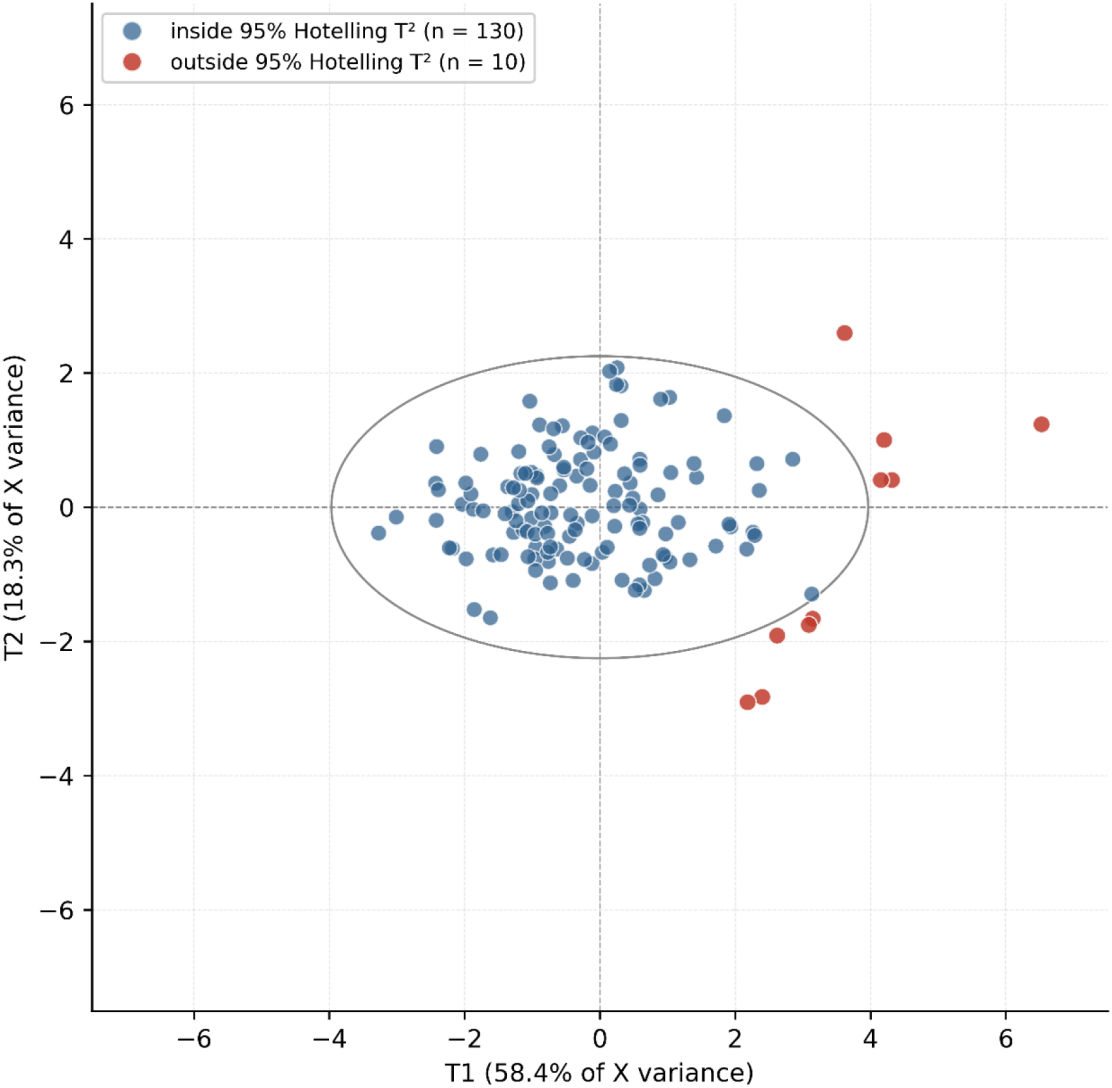
Scores plot (T1 vs T2) of the PLS model based on autoscaled mineral predictors and polyphenol responses (n = 140 cultivars).

**Figure 5:**
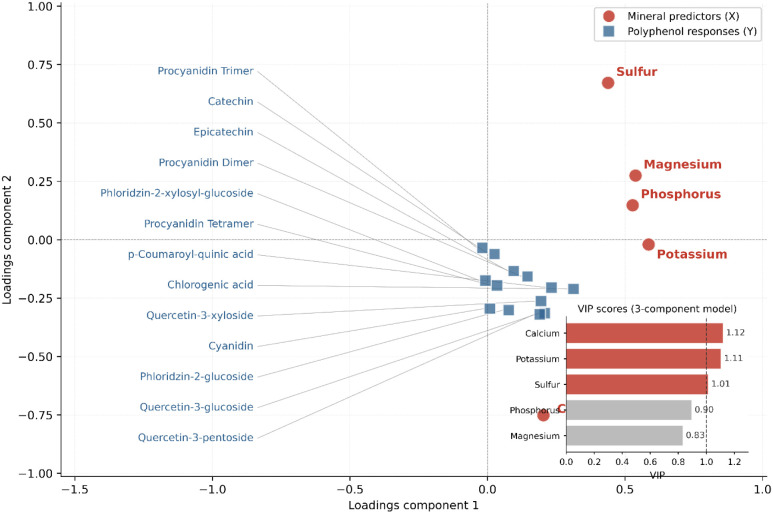
Loadings plot (component 1 vs 2) showing mineral predictors (circles) and polyphenol variables (squares) in the latent space of the fitted three-component PLS model. Inset: variable importance in projection (VIP) scores derived from the same three-component model; dashed line indicates VIP = 1. Given the low Q^2^/R^2^Y, VIP values are interpreted only as descriptive within-model prioritization.

Accordingly, the PLS analysis should be interpreted as hypothesis-generating and descriptive rather than predictive. The VIP ranking identifies variables that contribute most within the selected latent-variable structure, but the low Q^2^ and R^2^Y preclude strong claims that these minerals are dominant predictors of polyphenol composition. Calcium may receive a high VIP despite modest pairwise correlations because VIP reflects covariance with the multivariate Y-block across latent components, including the cyanidin-related signal, and because it is comparatively independent from the strongly correlated K/P/Mg/S mineral block.

### GAM Analysis: Nonlinear Response Patterns

Pairwise correlations and PLS regression mainly capture monotonic or predominantly linear structure and may therefore miss threshold-like or nonmonotonic motifs. In the present study, GAM/spline analysis was used as an exploratory visualization tool for two biologically and agronomically plausible candidate relationships, not as confirmatory evidence for nonlinearity across the full 5 × 13 mineral–polyphenol matrix. The primary statistical interpretation therefore remains based on FDR-corrected Pearson and Spearman correlations, supported by targeted robustness checks. Quartile-stratified correlations were used descriptively to illustrate possible range dependence; sign changes across quartiles were not treated as independent statistical proof of nonlinearity.

For sulfur and chlorogenic acid, the overall Pearson association was weak but FDR-significant, whereas the Spearman association was not significant after FDR correction, indicating that the relationship was not rank-robust. Nevertheless, exploratory spline modeling suggested a possible range-dependent pattern. In the Python-based GAM refit, the sulfur–chlorogenic acid smooth was fitted with a cubic B-spline basis dimension of k = 10 and had an effective degrees of freedom of 1.61. The smooth term remained statistically significant after FDR correction across the two reported GAM smooths (p = 0.0315; q = 0.0315), but the explanatory strength was low (pseudo R^2^ = 0.050). Thus, the model supports only a weak exploratory nonlinear motif rather than a strong predictive or causal relationship.

This sulfur–chlorogenic acid pattern is biologically interesting because sulfur participates in amino acid metabolism, redox regulation and stress-related metabolic processes. It is also agronomically relevant because the orchard was managed under a documented organic cultivation routine in which wettable sulfur was used as part of scab management. However, sulfur inputs were not experimentally varied as treatment factors, and protein content, cysteine/methionine pools, sulfur-containing secondary metabolites and cultivar-specific sulfur residue dynamics were not measured. Therefore, the present data cannot distinguish whether fruit sulfur variation reflects protein-related composition, redox metabolism, cultivar-specific uptake/allocation, plant-protection-related exposure, or a combination of these factors. The sulfur–chlorogenic acid pattern should therefore be interpreted as a mechanistic hypothesis for future controlled experiments with direct sulfur-exposure, residue-dynamic and protein/metabolite measurements (Figures 6 and 7).

**Figure 6:**
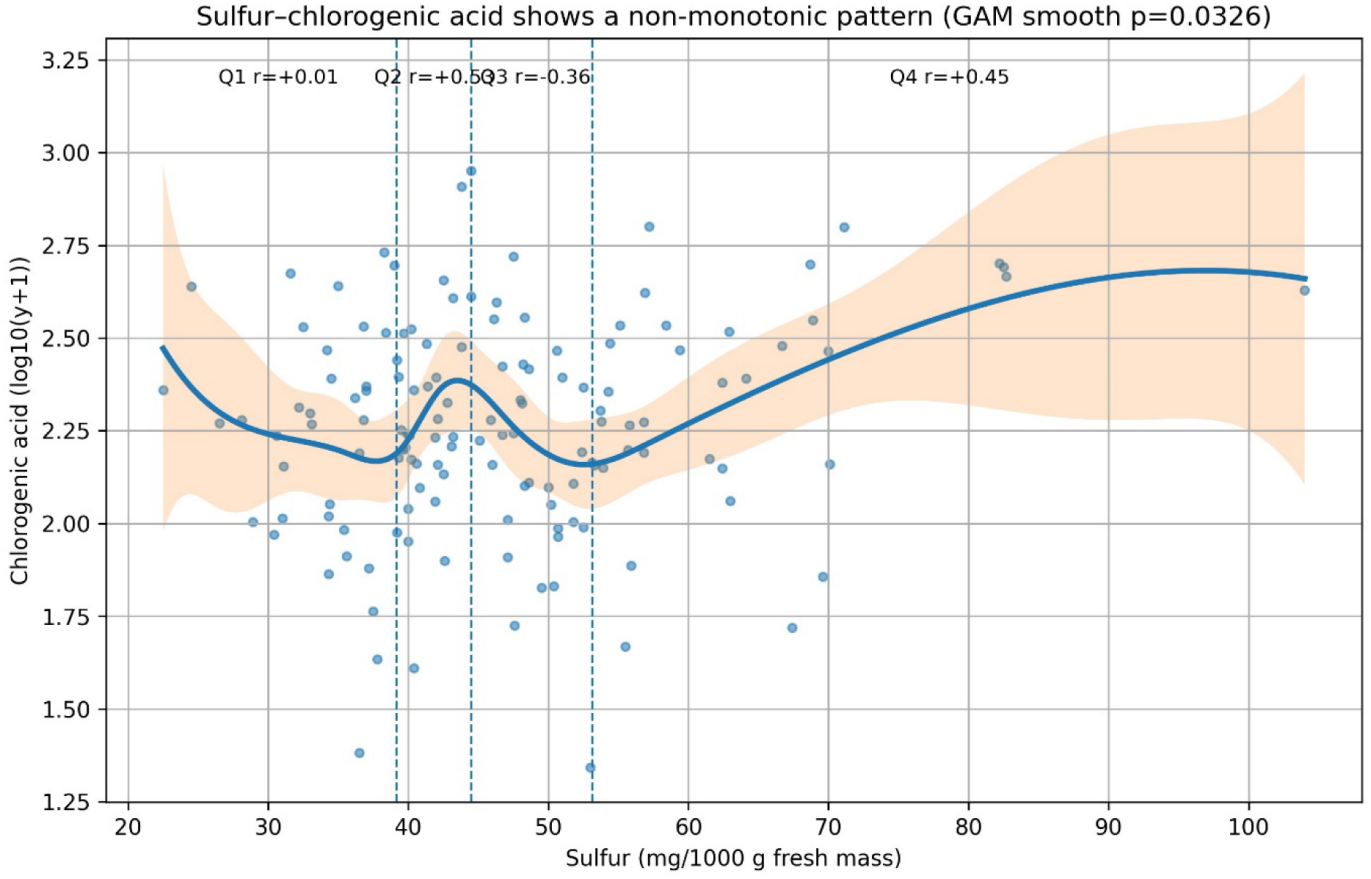
Exploratory sulfur–chlorogenic acid pattern across cultivars. Scatter plot of cultivar-specific chlorogenic acid concentration as a function of fruit sulfur content. The solid line shows the fitted exploratory Python-based GAM/spline smooth for log_10_(concentration +1)-transformed chlorogenic acid, with the shaded area indicating the 95% confidence band. A cubic B-spline basis dimension of k = 10 was used; the smooth had an effective degrees of freedom of 1.61 and was nominally supported after FDR correction across the two reported smooths (p = 0.0315; q = 0.0315), but explained only limited variation (pseudo R^2^ = 0.050). Vertical dashed lines mark sulfur quartile boundaries; within-quartile Pearson correlations (r) are annotated descriptively to illustrate possible range-dependent sign changes. The pattern is interpreted as an exploratory candidate association rather than as evidence of a causal sulfur response.

**Figure 7:**
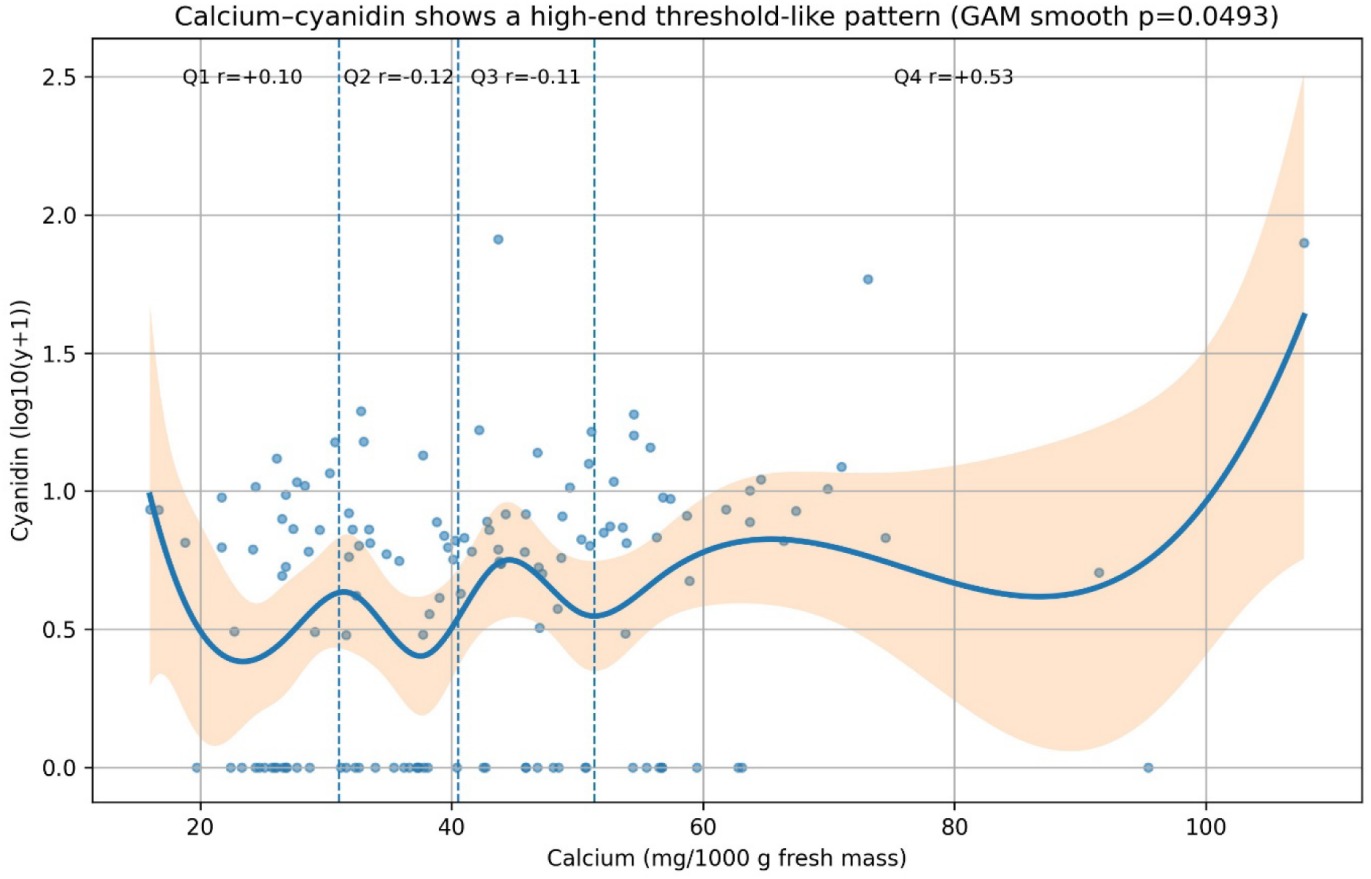
Exploratory calcium–cyanidin pattern across cultivars. Scatter plot of cultivar-specific cyanidin concentration as a function of fruit calcium content. The solid line shows the fitted exploratory Python-based GAM/spline smooth for log_10_(concentration +1)-transformed cyanidin, with the shaded 95% confidence band. A cubic B-spline basis dimension of k = 10 was used; the smooth had an effective degrees of freedom of 0.79 and was nominally supported after FDR correction across the two reported smooths (p = 0.0129; q = 0.0258), but explained only limited variation (pseudo R^2^ = 0.047). Vertical dashed lines indicate calcium quartile boundaries, and within-quartile Pearson correlations are annotated descriptively. Given the zero-inflated cyanidin distribution and sensitivity to high-cyanidin cultivars, the pattern should be interpreted as exploratory rather than as evidence of a general calcium-driven threshold response.

As a secondary exploratory example, calcium and cyanidin showed a positive Pearson correlation, but this pattern must be interpreted with particular caution because cyanidin was strongly zero-inflated (46/140 zero values). The Spearman association did not survive FDR correction (ρ = 0.19, p = 0.027, q = 0.083). In the Python-based GAM refit, the calcium–cyanidin smooth was fitted with a cubic B-spline basis dimension of k = 10 and had an effective degrees of freedom of 0.79. The smooth term was nominally supported after FDR correction across the two reported smooths (p = 0.0129; q = 0.0258), but explanatory strength was again low (pseudo R^2^ = 0.047). The low effective degrees of freedom indicate a strongly smoothed pattern rather than a complex, well-supported threshold curve.

Additional robustness checks further weakened the interpretation of the calcium–cyanidin association. A logistic model for cyanidin presence/absence using standardized calcium was not significant (p = 0.165), the Spearman association among nonzero cyanidin cultivars was not significant (ρ = 0.17, p = 0.095), and excluding the upper 5% of cyanidin values reduced the relationship to ρ = 0.14 (p = 0.113). Thus, the apparent calcium–cyanidin motif may reflect a subset of high-cyanidin cultivars or distributional features of the zero-inflated response variable rather than a general calcium response. Because calcium was supplied as part of the documented orchard-management routine but was not experimentally varied, this pattern should be considered exploratory and hypothesis-generating.

### Biological Interpretation

The combined evidence—robust but selective K/P associations with chlorogenic acid and quercetin glycosides, weak multivariate PLS coupling, and exploratory candidate nonlinear motifs—supports a cautious model in which fruit mineral status covaries with phenolic composition in a compound-specific manner. The phenylpropanoid pathway is tightly regulated at multiple levels (transcriptional control, enzyme activity, substrate channeling, transport, redox regulation), and its outputs often respond to nutrient status and stress signaling.^1,2,20,21^ Chlorogenic acid in particular is produced via hydroxycinnamoyl-CoA quinate transferase (HQT)-catalyzed transesterification of caffeoyl-CoA with quinic acid,^23^ and primary apple-specific work has shown that phenylpropanoid pools respond differentially to mineral nutrition.^25^ However, the present data do not demonstrate that mineral supply drives phenolic accumulation, because cultivar identity, uptake/allocation traits, maturity and microenvironmental variation remain potential contributors.

For sulfur specifically, the present data do not support a strong monotonic association with chlorogenic acid, because the Pearson association was weak and Spearman-inconsistent. Possible mechanistic interpretations include sulfur-related redox metabolism, sulfur-containing amino acid/protein pools, or management-related exposure, but none of these mechanisms can be resolved without direct measurements of protein, cysteine/methionine, glutathione, sulfur application history and sulfur speciation.^3,21,22^

### Implications for Cultivar Comparison and Future Orchard Experiments

The strongest practical implication of the present study concerns cultivar comparison rather than immediate orchard management. Mineral composition should be considered as a potential covariate when comparing polyphenol profiles among cultivars, particularly for K/P-associated phenolic-acid and quercetin-glycoside patterns. For breeding or germplasm evaluation, cultivars with high phenolic content across variable mineral backgrounds may be candidates for further stability testing.

The observed candidate response windows should not yet be translated into orchard recommendations. Instead, they define testable hypotheses for intervention trials in which sulfur supply, sulfur-based plant-protection regimes, calcium foliar applications, fruit maturity, protein content and redox markers are monitored directly. Such experiments would be required before mineral management could be linked causally to targeted changes in phenolic composition.

Taken together, this study provides a cultivar-scale view of how fruit mineral status and polyphenol profiles covary across a broad common-garden apple panel. The clearest and most reproducible result is the positive association of potassium and phosphorus with chlorogenic acid and related phenolic variables, whereas sulfur- and calcium-related patterns are weaker, more exploratory and sensitive to distributional features such as zero inflation. PLS confirmed that the mineral block contains some multivariate information about the polyphenol block, but low Q^2^ and R^2^Y show that fruit mineral composition alone has limited predictive power. The main contribution is therefore not an orchard-management prescription, but a reproducible association framework and a set of prioritized hypotheses for controlled mineral-supply, plant-protection and cultivar-stability experiments. These findings are specific to the present cultivar panel grown under one documented organic management regime and cannot be generalized across orchards, climates or management systems; any use for cultivar comparison or hypothesis prioritization should therefore be restricted to comparable settings.

### Limitations

Several limitations should be considered. First, although common-garden cultivation reduces environmental noise, cultivar-level mineral content still reflects genotype-specific uptake and allocation, and residual microenvironmental variation cannot be fully excluded.^8,9^ Second, the sampling design produced one homogenized composite per cultivar; analytical duplicates therefore quantify measurement precision but not within-cultivar biological variability among fruits, trees or seasons. Third, correlation, PLS and exploratory GAM/spline approaches identify association patterns but do not prove causality; controlled intervention trials are needed to test whether altering sulfur or calcium exposure reproducibly shifts chlorogenic acid or cyanidin concentrations. Fourth, cyanidin was zero-inflated, and calcium–cyanidin patterns were not robust to sensitivity checks. Fifth, sulfur and calcium inputs were not experimentally varied and were not resolved at cultivar-specific exposure level and protein content or sulfur-containing amino acids were not measured; sulfur-related interpretations are therefore mechanistic hypotheses rather than demonstrated pathways. Finally, polyphenol profiles are sensitive to maturity stage, peel proportion and postharvest handling, all of which should be controlled or modeled explicitly in future work.

Future work should combine controlled manipulation of sulfur and calcium regimes (soil supply vs foliar application vs fungicide-associated exposure), cultivar replication across seasons, independent tree-level replication, and mechanistic readouts such as protein content, cysteine/methionine, glutathione, redox markers, enzyme activity proxies and peel-specific profiling. Such designs are required to establish causal pathways and define whether mineral management can reproducibly modulate fruit phenolic composition.

## Supplementary Material



## Data Availability

The complete numerical data set required to reproduce the statistical analyses reported in this manuscript is provided as Supporting Information, with cultivars represented by anonymized cultivar IDs. To protect the integrity of an independently generated cultivar-identifiable data set while enabling academic verification, the cultivar ID–cultivar-name key is available from the corresponding author upon reasonable request for noncommercial academic research and reanalysis, subject to a data-use agreement. The key may not be redistributed or used for commercial purposes without prior written permission. Because the cultivar IDs are assigned in alphabetical order of cultivar names, publishing the names as a list would allow reconstruction of the cultivar-to-data mapping and is therefore not provided.

## Notes

Table S1: provides the numerical data set with cultivar IDs and concentrations of the 13 quantified polyphenol variables and 5 mineral elements; Table S2: provides the full mineral–polyphenol Pearson/Spearman correlation matrix with nominal p-values and FDR-adjusted q-values, PLS summary metrics, and sensitivity analyses; Table S3: provides within-block mineral–mineral and polyphenol–polyphenol correlation matrices; Table S4: shows partial correlations between each mineral and each polyphenol, controlling for the remaining four minerals, and complemented by ridge regression on autoscaled predictors (PDF)

## Abbreviations

Ca: calcium
CV: coefficient of variation
FDR: false discovery rate
FW: fresh weight
GAM: generalized additive model
HPLC-MS: high-performance liquid chromatography–mass spectrometry
ICP-OES: inductively coupled plasma–optical emission spectrometry
IQR: interquartile range
K: potassium
Mg: magnesium
P: phosphorus
PLS: partial least-squares
RMSE: root-mean-square error
S: sulfur
SD: standard deviation
VIP: variable importance in projection
