# Supplementary material for: Associations between Fruit Mineral Composition and Polyphenol Profiles across 140 Dessert Apple Cultivars (Malus domestica)
Source: J Agric Food Chem. 2026 Jul 15;74(29):22908–18. doi: 10.1021/acs.jafc.6c06394 (PMC13426305; doi:10.1021/acs.jafc.6c06394)
Supplement: Supplementary file 1 [file jf6c06394_si_001.pdf]

Supporting Information

Associations between fruit mineral composition and polyphenol profiles across 140 dessert apple cultivars (Malus domestica)

Eleonora Zickenheiner*

Humboldt-Universität zu Berlin, Faculty of Life Sciences,

Albrecht Daniel Thaer-Institute of Agricultural and Horticultural Sciences,

Invalidenstraße 42, 10115 Berlin, Germany

*Corresponding author: e.zickenheiner@hu-berlin.de

Supplementary Tables S1: numerical dataset with cultivar IDs and concentrations of the 13 quantified polyphenol variables and 5 mineral elements

Polyphenol and mineral content of 140 dessert apple varieties (mg/1000g fresh weight)

Cultivar Nr.	**Chlorogenicacid**	**p-Coumaroyl-quinic acid**	**Quercetin-3-glucoside**	**Quercetin-3-xyloside**	**Quercetin-3-pentoside**	**Catechin**	**Epicatechin**	**Procyanidin Dimer**	**Procyanidin Trimer**	**Procyanidin Tetramer**	**Phloredzin-2-xylosyl-glucoside**	**Phloredzin-2-glucoside**	**Cyanidin**	**Potassium**	**Sulphur**	**Phosphorus**	**Magnesium**	**Calcium**
1	66.10	92.40	8.13	11.29	9.11	290.26	137.99	176.67	159.60	154.46	58.49	65.22	0.00	1307.50	49.50	122.10	57.30	19.70
2	303.61	6.39	7.71	11.33	12.33	148.11	243.19	187.82	114.54	72.65	194.39	280.27	80.74	1127.20	41.30	102.20	51.40	43.70
3	74.69	42.09	6.53	11.13	8.11	587.49	472.06	240.46	258.55	207.07	42.04	54.25	3.75	1515.60	37.20	140.10	61.70	58.90
4	291.69	8.65	4.38	5.52	5.29	145.47	127.30	141.28	103.35	49.38	14.65	32.04	15.45	1522.40	50.60	127.00	65.60	51.10
5	231.54	32.43	9.91	13.13	10.77	198.63	155.62	149.79	59.64	40.77	73.03	37.28	0.00	1873.10	52.50	102.50	64.60	55.50
6	71.01	31.83	7.47	12.50	10.29	195.25	126.48	150.67	42.34	12.06	25.41	19.69	5.37	1317.60	69.60	132.50	69.80	32.60
7	489.83	407.97	13.57	19.91	16.72	362.37	667.86	481.08	294.73	214.47	245.07	168.65	15.63	2854.80	82.50	242.20	108.90	42.20
8	103.63	40.36	4.21	8.83	9.34	284.48	117.17	98.52	118.25	47.91	25.94	22.51	4.79	1141.70	34.30	77.80	39.20	31.80
9	80.79	101.26	5.31	7.12	6.28	639.13	168.88	112.87	135.64	79.01	14.37	47.28	2.06	1302.90	35.60	93.70	56.30	53.80
10	328.03	90.94	5.16	5.67	6.14	264.06	215.63	260.87	88.31	107.87	119.28	83.85	6.28	1998.30	62.90	194.30	68.50	32.10
11	267.78	5.29	2.62	3.40	3.98	286.37	28.94	26.86	9.21	46.91	164.06	38.44	0.00	1772.80	48.20	142.80	63.80	56.50
12	245.18	104.65	9.54	12.21	8.14	754.64	273.93	348.24	92.68	66.12	153.37	116.86	11.57	1871.90	64.10	176.60	71.40	50.90
13	96.64	55.98	3.75	5.15	6.22	251.99	209.87	174.60	180.25	138.69	122.93	87.20	5.27	1380.20	52.50	128.60	58.00	21.70
14	126.95	76.65	6.54	8.71	8.43	173.47	108.63	102.40	65.68	34.70	49.58	34.95	13.44	1299.70	51.80	92.00	67.90	55.80
15	172.28	30.38	3.53	5.76	5.07	497.12	259.70	163.38	187.56	52.28	46.27	175.82	6.73	1165.80	46.70	89.30	50.10	63.70
16	148.19	25.75	4.69	6.52	7.07	350.26	195.44	244.22	133.49	81.34	195.78	188.52	2.02	1462.30	61.50	133.30	54.90	31.60
17	42.05	30.90	4.00	5.08	5.12	152.52	150.70	153.68	63.63	90.51	66.31	41.16	9.20	1118.70	37.80	92.30	56.60	69.90
18	100.09	34.77	6.21	10.68	10.64	342.12	158.87	102.70	115.88	52.88	149.35	120.94	8.52	1432.00	51.80	115.30	57.40	21.70
19	404.18	214.47	7.15	10.08	15.13	370.48	606.99	248.38	53.89	17.71	127.41	98.93	0.00	1572.90	43.20	146.90	53.10	56.70
20	185.62	44.60	5.44	6.39	6.64	311.25	264.82	385.25	188.02	191.94	119.33	71.86	0.00	955.50	26.50	79.20	38.80	24.70
21	292.24	87.03	3.82	5.01	5.07	347.82	73.42	104.72	0.00	44.50	117.08	143.07	0.00	977.50	34.20	87.20	47.00	26.60
22	157.46	40.23	5.09	6.34	4.26	224.34	275.42	195.41	118.87	97.33	349.24	144.27	6.09	1524.30	39.70	108.80	56.10	52.10

23	170.06	131.03	4.70	6.03	5.49	481.91	327.95	17.48	15.90	59.87	141.04	89.02	6.26	1221.70	43.20	127.30	54.40	29.50
24	434.29	42.69	4.54	7.30	9.47	523.10	459.05	275.42	141.44	369.01	451.42	241.01	0.00	1751.80	24.50	106.10	55.50	31.60
25	140.21	5.71	4.29	6.48	7.48	386.62	193.61	84.20	132.48	60.82	147.81	84.69	5.90	1389.80	53.90	108.00	72.20	39.40
26	186.60	110.49	3.23	4.05	4.83	486.78	235.89	236.90	159.10	110.52	77.40	52.33	0.00	1324.80	56.80	118.10	58.10	42.70
27	352.89	116.30	8.50	10.00	12.39	586.13	190.25	245.15	242.26	292.44	39.02	56.11	7.26	2069.00	68.90	172.10	78.40	45.90
28	66.85	57.51	16.18	27.04	23.85	339.94	195.88	256.26	198.11	160.08	68.34	150.14	0.00	1727.60	50.40	146.60	57.10	27.70
29	246.49	157.77	8.55	20.24	23.78	584.00	956.75	338.27	78.28	65.97	219.73	182.60	4.73	1240.00	42.00	124.80	59.70	48.70
30	501.46	112.23	8.13	11.81	8.24	856.99	274.68	180.79	155.02	56.30	319.19	145.55	0.00	2449.00	82.20	207.50	74.70	54.40
31	76.12	62.84	4.92	5.74	5.36	223.99	136.12	97.35	27.64	52.38	76.39	51.66	5.49	1458.20	55.90	134.20	62.90	33.50
32	233.09	70.48	3.31	5.31	4.07	313.87	173.21	186.55	91.95	92.06	46.33	70.68	0.00	1237.10	41.40	94.30	53.20	33.90
33	145.00	49.73	5.58	7.85	6.35	337.14	172.70	269.62	173.17	82.10	60.88	44.54	7.55	1305.30	53.10	114.50	57.20	16.70
34	134.95	102.73	4.88	7.75	6.13	376.20	257.12	232.96	289.44	151.11	88.06	79.10	5.81	1028.40	42.50	96.20	51.40	56.30
35	275.08	104.46	10.90	12.88	12.67	251.98	133.89	221.87	166.61	114.94	38.68	97.53	4.09	1773.90	39.20	168.80	63.80	91.50
36	393.66	77.47	7.46	7.38	8.11	379.69	178.89	259.17	142.44	388.91	190.11	84.83	0.00	1939.90	46.30	129.50	56.70	32.60
37	341.13	72.33	13.10	21.65	24.01	287.20	334.59	301.87	236.07	119.57	27.88	94.58	14.04	1797.70	55.10	179.20	63.80	30.70
38	57.06	20.47	4.18	6.17	6.76	116.88	91.63	95.45	51.93	82.61	58.91	21.28	9.04	999.00	37.50	66.70	53.80	63.70
39	78.27	26.21	4.09	5.83	6.52	140.35	94.14	74.99	85.48	56.66	68.59	24.27	18.03	1107.90	42.60	89.90	58.50	54.50
40	341.40	6.08	7.13	9.65	11.26	175.36	126.80	256.67	152.71	28.04	342.00	124.61	5.28	1415.20	58.40	121.80	58.80	39.70
41	259.56	56.61	16.36	18.60	33.17	401.85	165.16	225.58	218.81	188.10	60.38	70.44	2.60	1391.40	48.60	113.40	60.60	38.20
42	523.86	7.28	5.15	7.23	10.00	490.15	223.15	224.01	78.79	123.30	75.39	85.45	0.00	1893.40	47.50	161.60	58.00	22.40
43	128.01	56.90	4.91	6.55	5.48	200.28	196.39	123.67	110.94	63.45	20.34	43.77	0.00	1466.00	48.60	124.40	69.00	48.50
44	536.49	296.02	6.42	12.52	9.37	717.75	989.44	182.21	281.90	81.74	314.78	264.41	2.20	1385.70	38.30	104.20	59.80	47.00
45	184.15	167.36	5.84	8.60	5.94	656.97	171.73	320.02	92.37	173.00	38.20	98.92	9.81	1375.60	33.10	121.40	47.60	27.70
46	228.10	48.11	18.68	26.57	29.75	181.24	152.71	291.06	118.84	127.81	66.74	155.00	78.08	1550.40	40.40	143.50	69.40	107.80
47	20.99	19.09	3.54	5.35	3.78	313.13	32.76	12.62	15.38	9.63	22.10	16.68	7.25	1489.00	53.00	129.40	59.40	44.30
48	211.04	94.80	8.64	10.04	22.24	273.59	439.98	291.95	229.82	179.64	62.34	73.89	18.50	1650.40	42.80	236.30	58.40	32.80
49	228.24	69.49	9.90	13.24	12.83	519.14	183.03	221.61	162.00	132.99	41.51	104.87	9.37	1240.90	22.50	111.40	40.90	24.40
50	39.74	4.64	3.89	4.35	6.50	351.73	218.25	202.74	156.66	70.12	108.06	27.59	0.00	1239.40	40.40	85.30	53.10	45.90
51	125.58	76.66	3.89	6.77	4.66	220.25	203.31	206.44	69.28	77.95	119.12	73.09	5.49	1601.80	48.30	110.20	66.60	53.90

52	113.96	76.13	6.19	11.31	7.83	567.22	261.68	186.02	210.00	99.74	110.05	106.55	0.00	1372.30	63.00	139.80	64.70	37.30
53	99.81	45.02	3.38	5.32	8.58	585.38	155.43	187.01	45.17	91.01	57.42	41.08	7.60	1268.00	28.90	102.30	44.80	16.00
54	264.05	134.41	4.57	6.16	10.29	306.84	690.44	227.74	165.93	104.80	24.42	101.55	4.32	1466.30	46.70	137.20	58.70	26.80
55	188.96	90.67	2.76	3.23	3.29	352.54	193.26	186.94	128.00	64.79	65.13	38.64	0.00	1316.00	45.90	122.30	59.60	26.90
56	148.00	40.11	3.84	6.49	5.81	326.49	191.71	176.46	65.81	137.84	312.07	93.45	7.17	1194.50	40.20	94.20	51.50	58.70
57	238.94	57.00	5.22	8.04	8.68	248.25	230.98	230.77	163.42	86.99	20.05	35.23	5.53	1574.60	62.40	140.60	65.50	18.80
58	187.10	84.82	8.34	12.16	9.79	175.21	139.48	113.78	124.55	43.66	288.75	143.75	5.62	1762.80	53.80	123.40	81.50	66.40
59	51.32	30.72	8.77	15.55	9.55	210.17	91.29	101.01	44.72	62.16	5.71	16.06	4.48	1106.00	67.40	134.10	73.60	43.90
60	102.13	4.47	4.36	4.93	5.20	122.90	119.04	142.62	101.67	33.36	20.57	27.01	6.25	1030.90	31.00	82.60	43.70	43.00
61	227.56	59.40	37.41	56.68	38.74	351.12	239.15	321.81	176.66	176.42	162.20	225.31	6.78	1760.30	37.00	154.90	57.20	42.80
62	72.18	27.18	5.88	8.39	10.57	380.19	60.89	95.81	63.20	35.89	43.85	26.19	10.65	1331.40	34.30	126.50	57.40	30.30
63	188.95	68.31	18.20	18.84	22.22	202.70	230.78	151.42	160.08	93.15	63.40	161.50	4.03	1076.60	36.80	113.60	54.80	47.20
64	197.64	6.10	2.43	2.39	2.63	227.53	141.38	175.66	94.04	73.69	44.46	7.00	6.47	1098.10	33.00	83.60	53.40	52.60
65	113.66	4.59	6.58	11.33	7.40	256.29	169.62	197.82	137.98	88.90	38.85	27.60	6.95	988.70	41.90	74.40	49.60	26.50
66	149.33	70.56	7.88	12.18	12.22	407.86	275.27	329.34	70.18	166.45	76.08	81.87	4.47	999.60	39.30	86.40	49.10	43.90
67	143.42	70.37	5.04	9.68	6.56	363.41	279.93	190.95	198.21	61.17	128.24	111.06	0.00	1422.00	70.10	140.20	62.00	25.70
68	174.01	157.42	3.87	5.87	4.45	273.99	138.36	180.11	103.39	104.57	77.07	88.56	4.60	1132.70	47.50	123.90	52.80	35.80
69	325.28	197.03	13.06	19.97	28.09	237.77	198.08	218.99	83.54	52.30	159.83	84.87	5.34	1438.70	38.40	84.60	57.30	51.00
70	470.82	94.64	6.86	9.66	9.58	634.09	265.30	300.59	356.57	165.28	668.28	440.51	0.00	1263.90	31.60	107.70	52.00	48.10
71	298.05	6.42	2.96	4.37	3.41	369.92	253.21	291.01	167.55	127.60	36.66	171.81	6.31	1018.60	43.80	109.50	50.40	27.40
72	628.51	129.24	6.19	8.14	8.50	352.35	234.06	200.43	151.93	82.20	67.56	52.51	0.00	2343.00	71.10	255.20	79.30	25.10
73	93.66	49.33	4.12	5.98	6.31	255.25	224.46	196.87	142.15	114.20	23.74	105.71	0.00	1461.50	39.20	99.20	58.30	24.40
74	629.78	210.09	17.26	26.58	26.75	660.88	363.52	493.94	73.81	150.69	107.50	224.66	8.40	1924.70	57.20	159.70	71.30	57.40
75	108.68	178.35	6.69	10.01	8.30	794.92	194.41	154.67	174.51	155.12	113.94	40.98	0.00	1447.20	40.00	125.10	46.00	32.30
76	52.07	43.21	3.61	4.02	4.95	219.08	123.93	145.66	60.75	31.36	32.75	20.30	0.00	1752.00	47.60	177.00	59.20	35.40
77	144.12	64.44	12.65	27.32	21.99	444.77	268.62	247.55	72.21	91.69	55.86	33.64	0.00	1419.70	40.60	124.80	60.20	63.10
78	354.65	174.60	4.41	4.91	2.97	767.44	264.35	318.25	337.97	138.71	34.58	133.95	5.17	1332.50	46.10	123.70	45.70	24.20
79	142.41	5.32	6.97	12.01	7.40	165.05	165.11	183.68	79.53	53.51	28.25	24.52	5.04	1266.10	53.30	115.70	52.70	45.80
80	139.88	36.92	5.91	8.73	7.05	377.89	173.38	171.19	127.11	107.70	111.66	99.69	2.11	1841.60	62.40	129.80	73.90	22.70

81	292.40	120.22	7.88	10.62	9.38	507.47	276.52	219.68	158.46	128.18	28.70	94.98	8.52	1997.60	59.40	167.50	61.10	56.80
82	425.03	84.16	8.64	13.57	10.63	490.01	201.25	244.63	86.18	41.43	28.35	75.53	3.11	2265.00	104.00	184.90	76.70	39.00
83	247.42	90.94	4.49	5.85	6.99	260.44	232.26	197.82	85.71	92.25	176.11	85.97	0.00	1207.60	39.30	111.40	58.90	28.70
84	159.58	125.34	4.62	9.27	7.08	161.51	109.66	183.48	68.54	149.04	110.43	96.75	12.52	1373.60	39.80	124.40	57.70	37.70
85	123.96	45.43	5.01	5.28	7.94	291.66	88.61	117.19	49.90	67.00	143.48	68.02	5.05	1395.80	40.80	126.70	54.60	41.60
86	111.74	95.95	6.54	12.52	12.09	834.14	364.56	304.12	258.98	119.46	406.39	299.99	3.27	930.50	34.40	50.80	32.00	40.70
87	141.58	5.68	8.72	8.54	16.11	329.75	113.10	163.94	55.89	36.40	24.59	23.73	12.80	1273.70	31.10	91.80	56.10	46.80
88	418.11	103.01	15.86	27.19	33.26	251.52	285.00	207.67	49.50	49.06	39.70	125.32	3.95	2025.40	56.90	149.40	65.50	26.50
89	246.21	50.25	10.12	10.45	7.33	353.85	142.33	191.90	110.20	167.43	29.18	44.84	0.00	1669.20	51.00	158.00	62.00	23.30
90	154.01	77.81	15.52	15.27	14.79	318.28	663.42	264.39	59.04	152.45	30.69	50.56	12.17	1587.10	36.50	164.10	56.50	26.10
91	171.61	61.13	12.87	19.87	18.55	454.79	418.20	250.79	100.02	17.81	108.09	64.33	14.16	1273.60	30.60	181.00	50.90	33.00
92	80.20	55.72	8.35	11.19	8.97	194.40	175.45	174.63	128.57	175.92	52.66	55.28	4.32	1104.30	47.10	108.60	54.10	46.90
93	233.21	82.64	13.11	19.41	16.43	239.62	893.15	437.43	238.53	148.77	262.38	124.86	5.79	1376.60	37.00	122.50	47.60	41.00
94	171.76	6.55	4.60	5.94	5.34	331.20	168.23	139.70	139.24	76.36	121.43	166.63	5.70	1137.10	40.10	110.30	53.30	50.30
95	216.92	96.82	6.00	6.80	6.97	366.30	180.07	174.88	188.13	191.02	28.99	88.38	5.17	1581.30	36.20	98.00	53.90	43.70
96	154.99	60.19	7.03	11.74	9.28	345.05	236.45	220.22	210.01	105.19	78.95	91.13	5.79	1512.80	52.40	111.70	67.60	74.50
97	408.14	274.06	11.06	15.83	29.03	392.18	210.29	173.85	49.72	105.92	65.76	73.65	10.01	2151.70	44.50	184.00	73.70	64.60
98	462.51	8.20	7.43	12.03	16.18	275.66	192.88	339.29	0.00	74.00	40.83	135.96	11.24	2077.10	82.70	200.20	82.20	71.00
99	451.06	143.23	8.93	9.47	12.15	389.30	169.10	211.09	149.75	69.27	121.70	250.73	0.00	2389.00	42.50	194.20	64.80	50.60
100	88.58	44.75	5.23	6.81	6.77	237.41	130.33	107.54	142.48	54.96	28.13	30.65	6.75	1234.50	40.00	96.30	54.40	38.80
101	214.13	4.78	2.85	3.26	4.27	221.31	181.47	150.48	85.56	172.11	219.19	121.03	7.12	1474.70	48.00	108.20	58.40	48.80
102	305.45	4.96	15.10	20.63	13.17	300.18	193.17	235.47	110.06	113.74	69.45	132.00	14.93	1696.30	54.40	115.60	57.50	54.50
103	95.14	52.95	5.35	10.11	9.44	444.44	121.50	129.79	210.66	78.32	48.19	17.68	8.71	1324.20	35.40	116.00	54.40	26.80
104	333.47	6.34	6.38	8.73	7.82	420.58	179.71	245.69	117.05	57.64	34.86	58.18	5.65	1048.80	40.20	102.40	45.90	40.30
105	177.64	83.19	13.34	19.02	17.00	241.01	171.50	215.83	141.78	107.26	50.37	71.27	9.83	1449.30	39.50	114.20	61.70	52.90
106	338.63	90.35	6.53	11.63	15.33	458.37	176.46	235.09	214.47	154.57	107.63	69.11	5.05	931.60	36.80	102.20	42.00	28.60
107	498.28	603.09	9.16	16.59	22.65	284.46	480.38	446.06	0.00	108.60	22.73	424.67	0.00	2144.20	68.70	120.30	72.90	95.40
108	101.37	52.95	4.88	6.45	8.92	185.89	123.10	184.77	44.24	60.31	75.05	46.99	2.76	1325.30	47.10	92.60	52.40	48.40

109	808.94	10.64	12.91	16.01	20.68	494.52	385.06	89.58	180.37	47.46	58.33	91.61	0.00	1781.40	43.80	138.10	62.20	50.70
110	182.74	132.67	10.97	16.62	17.05	260.57	94.69	79.88	10.74	31.81	92.24	140.29	7.35	1688.40	55.80	140.90	54.80	31.80
111	436.44	138.00	7.15	8.15	8.68	413.45	177.21	324.45	269.96	171.54	245.18	289.04	0.00	1630.10	35.00	154.60	62.90	42.50
112	169.56	62.28	4.87	6.90	8.13	210.63	252.60	201.03	80.24	72.33	57.46	81.62	6.41	1311.00	41.90	84.60	57.50	53.60
113	154.24	7.31	13.51	18.79	14.87	247.29	166.81	209.29	80.44	114.70	50.04	88.01	0.00	1650.00	56.80	131.00	71.20	45.90
114	166.48	56.14	5.67	9.96	14.68	257.06	246.49	192.58	221.49	122.56	192.00	89.58	2.03	1284.20	45.10	107.60	64.90	37.70
115	300.54	6.32	6.69	9.89	8.93	265.12	285.93	199.39	83.84	143.48	110.20	50.29	0.00	1426.60	66.70	171.40	67.60	31.20
116	357.71	7.58	6.64	9.39	10.33	262.44	152.37	247.49	36.34	75.42	192.36	161.77	0.00	1751.20	48.30	100.90	67.40	62.80
117	45.57	48.13	5.73	10.42	14.19	141.88	185.95	143.11	87.95	96.29	76.48	33.85	4.66	1705.50	55.50	111.20	67.90	40.10
118	143.05	116.09	4.28	7.36	5.27	226.80	162.66	174.85	32.72	39.20	22.07	60.96	0.00	1218.30	46.00	112.80	48.20	36.20
119	160.29	35.72	5.35	7.79	7.36	244.94	204.72	173.07	216.05	44.92	52.53	65.89	0.00	1605.00	43.10	109.90	66.80	40.40
120	892.33	225.46	25.54	32.25	41.64	365.48	298.38	302.42	80.50	209.85	98.45	213.08	7.50	2199.90	44.50	157.10	65.90	67.40
121	92.31	6.85	6.08	7.24	32.49	194.12	113.54	112.89	40.29	123.85	26.63	35.57	9.45	1209.90	30.40	110.40	54.00	28.30
122	91.28	57.32	4.55	8.00	7.34	258.13	200.26	150.27	104.14	100.00	190.60	83.75	0.00	1461.00	50.70	119.60	63.10	26.10
123	204.34	60.55	4.49	5.24	5.15	307.28	286.40	412.07	149.99	191.11	128.19	76.60	4.56	1006.90	32.20	84.40	42.10	43.80
124	142.89	44.66	6.56	8.74	8.14	417.62	172.34	182.98	138.35	110.07	151.40	113.28	4.92	1354.00	42.10	111.00	57.20	34.80
125	189.12	127.78	11.57	15.83	13.09	262.99	177.24	195.40	136.45	175.61	53.29	124.35	7.61	1062.30	28.10	73.90	43.90	61.80
126	124.25	45.57	6.13	8.40	7.10	435.54	176.98	199.48	90.81	95.30	43.77	27.38	0.00	1405.70	50.00	99.40	60.20	37.80
127	495.57	100.53	6.45	8.31	8.43	248.45	116.17	139.10	252.28	94.81	451.96	364.77	0.00	1495.30	39.00	125.90	60.40	46.80
128	290.18	8.46	6.34	7.21	8.97	144.15	205.05	254.88	94.51	92.56	196.05	110.07	0.00	1885.90	70.00	187.10	65.40	25.90
129	200.76	96.24	9.19	15.06	12.59	222.18	291.56	427.79	95.50	198.84	72.02	22.14	3.20	2125.20	53.70	211.70	56.50	32.40
130	173.92	120.52	8.51	13.31	14.65	262.00	80.74	115.92	86.07	39.87	43.41	87.21	6.29	1205.10	39.90	101.60	55.00	33.40
131	190.31	64.36	10.16	21.58	12.38	441.34	204.44	160.44	133.45	117.20	104.33	121.03	0.00	1693.90	42.10	127.00	53.70	56.70
132	324.78	5.04	5.76	3.63	4.35	427.02	236.02	305.35	0.00	205.98	117.16	80.04	0.00	995.60	39.70	96.30	48.30	37.20
133	156.66	78.36	4.83	5.93	7.47	319.65	192.16	258.87	98.14	98.93	169.80	78.79	0.00	1834.90	55.70	138.30	74.40	59.50
134	96.03	32.61	9.03	14.60	8.96	177.43	141.42	115.25	75.23	60.69	34.91	32.13	9.34	1609.70	50.70	108.80	65.30	49.40
135	244.63	78.87	10.15	13.18	8.26	343.73	360.17	357.96	132.42	94.86	439.82	202.82	57.66	1052.40	34.50	71.90	42.70	73.10
136	23.07	14.67	6.90	9.61	9.20	333.39	192.24	188.47	122.49	84.20	35.21	93.58	2.09	1098.80	36.50	86.50	51.10	29.10
137	209.86	77.01	2.43	2.86	2.78	219.69	123.57	83.36	173.63	48.22	187.59	117.95	0.00	1351.50	48.10	112.90	58.90	26.80

138	337.25	99.32	8.27	15.06	20.62	395.80	250.50	298.02	240.95	215.71	539.90	446.10	0.00	1535.70	32.50	95.30	55.70	37.40
139	225.60	54.59	12.54	19.63	20.44	322.24	264.17	340.43	75.23	133.37	87.17	181.69	0.00	1359.40	54.30	117.00	64.60	36.60
140	111.27	47.24	3.57	4.62	4.37	347.34	177.26	276.36	77.68	110.89	106.11	49.26	0.00	1111.20	50.20	99.20	56.80	38.10

Supplementary Tables S2, S3: Full Pearson and Spearman matrices

Table S2: All FDR-significant Pearson mineral–polyphenol correlations (q < 0.05), sorted by |r|

Mineral	Polyphenol	Pearson r	Pearson FDR q	Spearman ρ	Spearman FDR q	Rank-robust
K	chlorogenic acid	+0.550	1.23×10^{-10}	+0.422	1.31×10^{-5}	yes
P	chlorogenic acid	+0.434	2.67×10^{-6}	+0.344	5.24×10^{-4}	yes
K	p-Coumaroyl-quinic acid	+0.391	3.99×10^{-5}	+0.243	0.025	yes
K	quercetin-3-glucoside	+0.336	7.90×10^{-4}	+0.371	2.06×10^{-4}	yes
Ca	cyanidin	+0.319	0.002	+0.187	0.083	no
K	quercetin-3-pentoside	+0.314	0.002	+0.339	5.43×10^{-4}	yes
K	quercetin-3-xyloside	+0.310	0.002	+0.315	0.002	yes
Mg	chlorogenic acid	+0.302	0.002	+0.207	0.055	no
P	quercetin-3-glucoside	+0.290	0.004	+0.361	2.61×10^{-4}	yes
P	p-Coumaroyl-quinic acid	+0.268	0.009	+0.289	0.004	yes
P	quercetin-3-pentoside	+0.259	0.011	+0.295	0.003	yes
Mg	p-Coumaroyl-quinic acid	+0.259	0.011	+0.091	0.455	no
P	procyanidin dimer	+0.255	0.012	+0.200	0.061	no
P	quercetin-3-xyloside	+0.252	0.012	+0.301	0.003	yes
K	procyanidin dimer	+0.245	0.015	+0.180	0.098	no
Ca	quercetin-3-glucoside	+0.232	0.023	+0.206	0.055	no
Ca	quercetin-3-xyloside	+0.229	0.025	+0.200	0.061	no
Ca	p-Coumaroyl-quinic acid	+0.222	0.030	+0.035	0.849	no
Ca	phloridzin-2-glucoside	+0.217	0.034	+0.120	0.324	no
S	chlorogenic acid	+0.212	0.038	+0.114	0.332	no
Ca	quercetin-3-pentoside	+0.210	0.039	+0.130	0.295	no
P	epicatechin	+0.202	0.049	+0.213	0.052	no

Table S3. Complete correlation matrix

Complete mineral–polyphenol correlation matrix across all 65 mineral–polyphenol combinations

Mineral	Polyphenol	Pearson r	Pearson p	Pearson FDR q	Pearson q<0.05	Spearman rho	Spearman p	Spearman FDR q	Spearman q<0.05
K	chlorogenic acid	0.550277	1.89E-09	1.23E-07	TRUE	0.422344	2.02E-04	1.31E-02	TRUE
K	p-Coumaroyl-quinic acid	0.390517	1.84E-03	3.99E-02	TRUE	0.243314	0.003770	0.024505	TRUE
K	quercetin-3-glucoside	0.336277	4.86E-02	7.90E-01	TRUE	0.371175	6.34E-03	2.06E-01	TRUE
K	quercetin-3-xyloside	0.310078	1.93E-01	0.001790	TRUE	0.315261	1.48E-01	0.001606	TRUE
K	quercetin-3-pentoside	0.314213	1.56E-01	0.001694	TRUE	0.339013	4.18E-02	5.43E-01	TRUE
K	catechin	0.115130	0.175565	0.330718	FALSE	0.078035	0.359437	0.507900	FALSE
K	epicatechin	0.151417	0.074125	0.160954	FALSE	0.194074	0.021584	0.070147	FALSE
K	procyanidin dimer	0.244847	0.003550	0.015383	TRUE	0.180044	0.033284	0.098340	FALSE
K	procyanidin trimer	0.004781	0.955293	0.955293	FALSE	0.004089	0.961756	0.980207	FALSE
K	procyanidin tetramer	0.151334	0.074286	0.160954	FALSE	0.080265	0.345826	0.499527	FALSE
K	phloridzin-2-xylosyl-glucoside	0.040779	0.632382	0.747360	FALSE	0.072673	0.393495	0.544195	FALSE
K	phloridzin-2-glucoside	0.172701	0.041305	0.099437	FALSE	0.210415	0.012583	0.054527	FALSE
K	cyanidin	-0.086954	0.306984	0.464045	FALSE	-0.115742	0.173268	0.331248	FALSE
S	chlorogenic acid	0.212397	0.011756	0.038206	TRUE	0.114324	0.178630	0.331741	FALSE
S	p-Coumaroyl-quinic acid	0.147211	0.082621	0.173238	FALSE	0.001730	0.983818	0.983818	FALSE
S	quercetin-3-glucoside	0.028866	0.734946	0.838097	FALSE	0.091857	0.280406	0.454939	FALSE
S	quercetin-3-xyloside	0.063831	0.453704	0.621537	FALSE	0.108424	0.202255	0.345963	FALSE
S	quercetin-3-pentoside	-0.017398	0.838338	0.879829	FALSE	0.010440	0.902567	0.980207	FALSE
S	catechin	-0.017159	0.840521	0.879829	FALSE	-0.117891	0.165370	0.325728	FALSE
S	epicatechin	-0.009622	0.910169	0.924390	FALSE	0.055500	0.514853	0.682968	FALSE
S	procyanidin dimer	0.091918	0.280086	0.441186	FALSE	0.017059	0.841442	0.959539	FALSE
S	procyanidin trimer	-0.107647	0.205530	0.351564	FALSE	-0.125047	0.140995	0.295634	FALSE
S	procyanidin tetramer	-0.160453	0.058255	0.135235	FALSE	-0.165529	0.050641	0.143115	FALSE
S	phloridzin-2-xylosyl-glucoside	-0.121743	0.151887	0.308520	FALSE	-0.027230	0.749457	0.902124	FALSE

S	phloridzin-2-glucoside	-0.063799	0.453930	0.621537	FALSE	-0.009616	0.910218	0.980207	FALSE
S	cyanidin	-0.102157	0.229734	0.382890	FALSE	-0.126620	0.136025	0.294720	FALSE
P	chlorogenic acid	0.434391	8.21E-05	2.67E-03	TRUE	0.343637	3.23E-02	5.24E-01	TRUE
P	p-Coumaroyl-quinic acid	0.267941	0.001371	0.008913	TRUE	0.289273	5.27E-01	0.003809	TRUE
P	quercetin-3-glucoside	0.290055	5.09E-01	0.003672	TRUE	0.360623	1.20E-02	2.61E-01	TRUE
P	quercetin-3-xyloside	0.252174	0.002649	0.012300	TRUE	0.300924	3.03E-01	0.002813	TRUE
P	quercetin-3-pentoside	0.258784	0.002020	0.011064	TRUE	0.295181	3.99E-01	0.003244	TRUE
P	catechin	0.119020	0.161326	0.317764	FALSE	0.152863	0.071374	0.193303	FALSE
P	epicatechin	0.202036	0.016673	0.049262	TRUE	0.213509	0.011312	0.052521	FALSE
P	procyanidin dimer	0.254633	0.002397	0.011985	TRUE	0.199880	0.017897	0.061281	FALSE
P	procyanidin trimer	0.049896	0.558244	0.693056	FALSE	0.014542	0.864595	0.968942	FALSE
P	procyanidin tetramer	0.095486	0.261759	0.425358	FALSE	0.068661	0.420200	0.569020	FALSE
P	phloridzin-2-xylosyl-glucoside	-0.073398	0.388779	0.574333	FALSE	0.004437	0.958507	0.980207	FALSE
P	phloridzin-2-glucoside	0.016230	0.849049	0.879829	FALSE	0.128994	0.128776	0.294720	FALSE
P	cyanidin	-0.015827	0.852757	0.879829	FALSE	-0.046195	0.587832	0.764182	FALSE
Mg	chlorogenic acid	0.301718	2.92E-01	0.002368	TRUE	0.207530	0.013880	0.055151	FALSE
Mg	p-Coumaroyl-quinic acid	0.258515	0.002043	0.011064	TRUE	0.090692	0.286577	0.454939	FALSE
Mg	quercetin-3-glucoside	0.187267	0.026722	0.070892	FALSE	0.239168	0.004428	0.026167	TRUE
Mg	quercetin-3-xyloside	0.186614	0.027266	0.070892	FALSE	0.230095	0.006240	0.033799	TRUE
Mg	quercetin-3-pentoside	0.187625	0.026428	0.070892	FALSE	0.220101	0.008974	0.044869	TRUE
Mg	catechin	-0.113606	0.181390	0.330718	FALSE	-0.132300	0.119182	0.294720	FALSE
Mg	epicatechin	0.061756	0.468543	0.621537	FALSE	0.038605	0.650651	0.829261	FALSE
Mg	procyanidin dimer	0.058863	0.489675	0.627247	FALSE	-0.020269	0.812111	0.942629	FALSE
Mg	procyanidin trimer	-0.058528	0.492148	0.627247	FALSE	-0.090620	0.286962	0.454939	FALSE
Mg	procyanidin tetramer	-0.049030	0.565107	0.693056	FALSE	-0.112470	0.185823	0.335513	FALSE
Mg	phloridzin-2-xylosyl-glucoside	-0.039400	0.643945	0.747436	FALSE	0.003728	0.965127	0.980207	FALSE
Mg	phloridzin-2-glucoside	0.019140	0.822406	0.879829	FALSE	0.028644	0.736912	0.902124	FALSE

Mg	cyanidin	-0.020530	0.809738	0.879829	FALSE	-0.086154	0.311474	0.470833	FALSE
Ca	chlorogenic acid	0.176060	0.037455	0.093636	FALSE	0.128026	0.131695	0.294720	FALSE
Ca	p-Coumaroyl-quinic acid	0.222103	0.008354	0.030166	TRUE	0.035264	0.679143	0.848929	FALSE
Ca	quercetin-3-glucoside	0.232296	0.005748	0.023352	TRUE	0.206388	0.014424	0.055151	FALSE
Ca	quercetin-3-xyloside	0.229364	0.006411	0.024513	TRUE	0.199852	0.017913	0.061281	FALSE
Ca	quercetin-3-pentoside	0.210526	0.012536	0.038802	TRUE	0.129476	0.127342	0.294720	FALSE
Ca	catechin	-0.090974	0.285074	0.441186	FALSE	-0.108811	0.200641	0.345963	FALSE
Ca	epicatechin	0.041045	0.630160	0.747360	FALSE	0.021874	0.797543	0.942551	FALSE
Ca	procyanidin dimer	0.108931	0.200141	0.351564	FALSE	0.006635	0.937988	0.980207	FALSE
Ca	procyanidin trimer	-0.113148	0.183167	0.330718	FALSE	-0.140424	0.097958	0.254691	FALSE
Ca	procyanidin tetramer	-0.061887	0.467599	0.621537	FALSE	-0.086665	0.308600	0.470833	FALSE
Ca	phloridzin-2-xylosyl-glucoside	0.071903	0.398542	0.575671	FALSE	0.083713	0.325432	0.480751	FALSE
Ca	phloridzin-2-glucoside	0.217321	0.009902	0.033876	TRUE	0.119569	0.159390	0.323762	FALSE
Ca	cyanidin	0.319080	1.22E-01	0.001583	TRUE	0.187249	0.026737	0.082758	FALSE

Supplementary Table S4

Independent contribution of individual minerals to polyphenol variation across 140 dessert apple cultivars.

(a) Partial correlations between each mineral and each polyphenol, controlling for the remaining four minerals (n = 140). Zero-order Pearson r is shown for comparison. p-values < 0.001 are given in scientific notation; FDR q-values are Benjamini–Hochberg-adjusted across the 30 partial-correlation tests reported here. Rows shaded in green remain significant after FDR correction and therefore represent associations that are independent of the shared mineral covariance structure.

Polyphenol	Mineral	Zero-order r	Partial r	p	FDR q
**chlorogenic acid**	**K**	+0.550	**+0.388**	2.18×10^{-6}	**6.53×10^{-5}**
	**P**	+0.434	+0.078	0.360	0.514
	**S**	+0.212	−0.062	0.467	0.592
	**Mg**	+0.302	−0.152	0.073	0.137
	**Ca**	+0.176	+0.145	0.087	0.154
**p-Coumaroyl-quinic acid**	**K**	+0.391	**+0.252**	0.003	**0.020**
	**P**	+0.268	+0.002	0.979	0.979

Polyphenol	Mineral	Zero-order r	Partial r	p	FDR q
	**S**	+0.147	−0.061	0.473	0.592
	**Mg**	+0.259	−0.035	0.681	0.757
	**Ca**	+0.222	+0.161	0.057	0.129
**quercetin-3-glucoside**	**K**	+0.336	+0.170	0.044	0.129
	**P**	+0.290	+0.157	0.065	0.129
	**S**	+0.029	−0.184	0.030	0.100
	**Mg**	+0.187	−0.031	0.720	0.772
	**Ca**	+0.232	+0.209	0.013	0.057
**quercetin-3-xyloside**	**K**	+0.310	+0.157	0.064	0.129
	**P**	+0.252	+0.108	0.205	0.307
	**S**	+0.064	−0.112	0.187	0.295
	**Mg**	+0.187	−0.038	0.656	0.757
	**Ca**	+0.229	+0.201	0.017	0.064
**quercetin-3-pentoside**	**K**	+0.314	+0.166	0.051	0.129
	**P**	+0.259	+0.129	0.127	0.212
	**S**	−0.017	**−0.254**	0.002	**0.020**
	**Mg**	+0.188	+0.043	0.610	0.732
	**Ca**	+0.211	+0.159	0.061	0.129
**cyanidin**	**K**	−0.087	−0.214	0.011	0.056
	**P**	−0.016	+0.216	0.010	0.056
	**S**	−0.102	−0.066	0.441	0.592
	**Mg**	−0.021	+0.011	0.896	0.927
	**Ca**	+0.319	**+0.364**	1.01×10^{-5}	**1.51×10^{-4}**

(b) Ridge-regression standardized coefficients for each polyphenol (response) modelled on all five minerals simultaneously (predictors and responses autoscaled; ridge penalty α = 1.0). Coefficients with |β| ≥ 0.3 are highlighted. Potassium dominates the chlorogenic-acid and p*-Coumaroyl-quinic acid, whereas calcium dominates the cyanidin model, consistent with the partial-correlation analysis.*

Polyphenol (response)	K	P	S	Mg	Ca
chlorogenic acid	**+0.641**	+0.116	−0.078	−0.217	+0.131
p-Coumaroyl-quinic acid	**+0.443**	+0.009	−0.084	−0.053	+0.163
quercetin-3-glucoside	+0.294	+0.243	−0.252	−0.048	+0.210
quercetin-3-xyloside	+0.278	+0.172	−0.157	−0.061	+0.208
quercetin-3-pentoside	+0.287	+0.202	**−0.355**	+0.070	+0.160
cyanidin	**−0.371**	**+0.337**	−0.091	+0.016	**+0.391**

Interpretation. After removing the shared mineral covariance, potassium is the only mineral with an independent, FDR-significant association with chlorogenic acid (partial r = 0.39) and p-Coumaroyl-quinic acid (partial r = 0.25), while the zero-order associations of phosphorus, sulfur and magnesium are largely explained by their collinearity with potassium. Calcium retains an independent association with cyanidin (partial r = 0.36); however, as detailed in the main text, the calcium–cyanidin relationship remains sensitive to the zero-inflated cyanidin distribution and to high-cyanidin cultivars and is therefore interpreted as exploratory. Analyses were performed in Python using scipy.stats (partial correlations via linear residualization) and scikit-learn (ridge regression).
